# Investigation on Variables Contributing to the Synthesis of C-S-H/PCE Nanocomposites by Co-Precipitation Method

**DOI:** 10.3390/ma14247673

**Published:** 2021-12-12

**Authors:** Ziyang You, Jing Xu

**Affiliations:** Key Laboratory of Advanced Civil Engineering Materials of Ministry of Education, Tongji University, Shanghai 201804, China; 1930651@tongji.edu.cn

**Keywords:** calcium silicate hydrate, nanocomposites, co-precipitation, synthetic variable, particle size distribution

## Abstract

The usage of nanoscale calcium silicate hydrate (nano C-S-H) proved to have an excellent promotion effect on the early performance of concrete as nano C-S-H with ultra-fine particle size can act as seeding for cement hydration. Therefore, it is of importance to tune the particle size during the synthesis process of nano C-S-H. In this paper, the influence of several variables of the particle size distribution (PSD) of nano C-S-H synthesized by chemical co-precipitation method with the aid of polycarboxylate (PCE) was studied by orthogonal experimental design. In addition, the composition, microstructure, and morphology of the C-S-H/PCE nanocomposites were analyzed by X-ray diffraction (XRD), scanning electron microscopy (SEM), transmission electron microscopy (TEM), and Raman spectrum. The results showed that the concentration of reactants had a significant impact on the PSD of C-S-H/PCE nanocomposites, followed by the dosage of dispersant. Ultrasonic treatment was effective in breaking the C-S-H/PCE aggregates with unstable agglomeration structures. The change in synthetic variables had a negligible effect on the composition of the C-S-H/PCE nanocomposites but had a significant influence on the crystallinity and morphology of the composites.

## 1. Introduction

With the rapid development of nanotechnology, which involves manipulating materials at scales around 100 nm or below, there has been a growing interest in the use of nanomaterials in construction materials, especially concrete [1,2,3,4,5], in recent years. A reduction in the particle size to the nanoscale has a pronounced impact on material performance. Many effects of the nanoparticles are associated with the high surface areas the particles provide. Nanoparticles may act as heterogeneous nucleation sites, and the addition of nanoparticles usually accelerates cement hydration. The promotion of cement hydration at a very early age is conducive to cementitious systems with supplementary cementitious materials (SCMs). This is of great significance for the alternatives to ordinary Portland cement (OPC) in the background of the reduction in CO_2_ emissions worldwide [6]. Among all types of nanoparticles, synthetic nano C-S-H has become one of the most attractive nanomaterials since a faster C-S-H formation, which contributes primarily to the strength development of cement paste, can be stimulated effectively by nano C-S-H nucleus [7,8,9]. Therefore, seeding by nano C-S-H has been considered as a promising strategy for many applications in concrete, while research interests on the synthesis of nano C-S-H have also begun to rise.

To date, a variety of methods, including mechanochemical [10,11,12], hydrothermal [13,14,15,16,17], sol–gel [11,18,19,20], and chemical co-precipitation [21,22,23,24,25,26,27], were developed to synthesize C-S-H. Both the mechanochemical and the hydrothermal methods are referred to as pozzolanic reaction systems, which produce C-S-H by the chemical reaction between calcium hydroxide and silicon dioxide in the presence of water [7]. For mechanochemical treatment, reactants are mixed in a mill, and the reaction products are formed during grinding. Although the reaction rate can be improved by milling, requirements on the mechanochemical equipment are very high. For the hydrothermal method, pozzolanic reactions take place under high temperatures and pressure in a sealed autoclave. It is relatively favorable for the synthesis of crystalline C-S-H. However, the reaction time and the energy demand are high. The sol–gel method involves a polycondensation reaction of metal alkoxides with water to be completed from a few minutes to a few hours. Common reactants are tetraethyl orthosilicate and calcium acetate. Although the reaction time of sol–gel method is quite short, it is still limited at the lab scale due to a complicated operation procedure and high requirements on the chemical reagents and equipment. Chemical co-precipitation is a simple, fast, and cheap method to synthesize C-S-H. The precipitation of C-S-H occurs immediately by mixing siliceous solution (such as Na_2_SiO_3_) and calcium salt solution (such as CaCl_2_ or Ca(NO_3_)_2_). The main disadvantage of the co-precipitation method is that too many variables could affect the reaction. In addition, it is also a challenge to control the particle size of the final products. Nevertheless, the chemical co-precipitation method is still competitive compared to others for its operation in ease, time-saving, and low cost.

Earlier works primarily focused on the effect of synthetic factors, such as initial calcium to silicon ratio (Ca/Si) [23,28,29,30,31,32], reaction temperature [22,33,34,35], pH [21], and reaction time [25], on the properties of C-S-H prepared by co-precipitation method. The initial Ca/Si ratio is one of the most extensively studied factors. In most cases, the initial Ca/Si ratio varied in the range of 0.6~2, and the variation in Ca/Si ratio could affect the crystallinity, degree of polymerization, particle size, morphology, and purity of synthesized nano C-S-H to a large extent, according to the literature survey. The temperature for C-S-H synthesis by co-precipitation usually varies between 20 °C and 60 °C. The variation in temperature would affect the particle size, degree of polymerization, and carbonization of C-S-H. By using a Response Surface Methodology, Sun et al. [22] obtained the minimum particle size with an optimal temperature of 30.1 °C. The preparation of C-S-H by the co-precipitation method should be carried out in an alkaline environment. The pH selected in the earlier researches ranged from 8.5 to 13.3. Kanchanason et al. [21] proved that the pH value had a significant impact on the composition, structure, morphology, and particle size of the synthesized C-S-H. Generally, the reaction speed of the co-precipitation method is relatively fast, but the reaction time is prolonged if dispersant or other organic polymers are added [7]. Most of the studies set the reaction time from a few hours to a few days. The change in reaction time affects the purity of products. Plank et al. [25] showed that the morphology of C-S-H could be altered during the reaction process.

Bone, tooth, and shell are natural inorganic-organic composites with excellent properties. Inspired by these natural materials, some latest works focused on the synthesis of C-S-H/polymer nanocomposites [24,28,36,37,38,39,40], one of which aims to regulate the particle size of C-S-H and create nano-sized accelerators for cement hydration. The most attractive polymer used is PCE, which is a well-known superplasticizer. It was found that both the features (such as chain length, side chains, charge density) and the dosage of PCE would profoundly affect nucleation growth, particle size, and the dispersion state of the synthesized nanocomposites [22,24,25,37,41].

Although several factors affecting the properties of synthetic C-S-H were intensively studied, as mentioned earlier, the influence of some other factors, such as the feeding order and speed of reactants and the concentration of reactant solution, on the properties of C-S-H are missing. Besides, the effect of the amount of PCE on the performance of synthetic products, especially the particle size, is worth studying. Therefore, this paper aims at synthesizing C-S-H/PCE nanocomposites by using the co-precipitation method, considering the feeding sequence and flow rate of reactants, the concentration of reactant solution, and the dosage of PCE as the primary synthetic variables, which could have a potential impact on the particle size of C-S-H. In addition, most of the existing researches only changes a certain synthesis factor (such as Ca/Si, PH, or the type of PCE) to study the properties difference of the synthesized C-S-H. Sun et al. [22] selected temperature, reactant flow velocity, and initial volume of PCE solution as synthesis variables and optimized the size of the synthesized C-S-H by response surface methodology. Similar systematic studies on synthetic variables affecting the performance of C-S-H are scarce. In order to remedy this deficiency, an orthogonal design was used to establish a relationship between the primary synthetic variables and the particle size of the nanocomposites. The effects of these variables on the composition, microstructure, and morphology of nano C-S-H/PCE were also studied.

## 2. Materials and Methods

### 2.1. Raw Materials

Calcium nitrate tetrahydrate (Ca(NO_3_)_2_·4H_2_O, AR, Purity: >99%) and sodium metasilicate nonahydrate (Na_2_SiO_3_·9H_2_O, AR, Purity(calculated as Na_2_O): 19.3~22.8%), which were purchased from Sinopharm Chemical Reagent Co. Ltd., were used for the synthesis of C-S-H/PCE nanocomposites. Sodium hydroxide (NaOH) and nitric acid (HNO_3_) purchased from the same supplier were used to adjust the pH during the synthesis. A mother liquor of HPEG-PCE with 45% solid content provided by Hangzhou Bailang Assistant Co. Ltd. was used as a dispersant and template during the synthesis of C-S-H.

### 2.2. Orthogonal Experimental Design

Four variables contributing to the synthesis of C-S-H/PCE nanocomposites, including feeding sequence of reactants, the flow rate of reactants, concentration of reactant solution, and the dosage of PCE, were studied. The dosage of PCE refers to the weight percentage of Ca(NO_3_)_2_·4H_2_O. Four levels were selected for each variable (Table 1). An L_16_(4^5^) orthogonal table was employed for the experimental arrangement (Table 2).

### 2.3. Synthesis of C-S-H/PCE Nanocomposites

C-S-H/PCE nanocomposites were synthesized by the chemical co-precipitation method. The initial molar ratio of Ca/Si in each group was 1.5, which is commonly used in previous studies. The reaction temperature was selected as 30 °C according to the optimization results of Sun et al. [22]. The pH was set at 11.6 by referring to Kanchanason et al. [21] and Sun et al. [22]. The general synthetic procedure of each group is as follows. First, Ca(NO_3_)_2_ solution, Na_2_SiO_3_ solution, and PCE solution were prepared according to the concentration set by the experimental design, while 1 mol/L HNO_3_ solution and 30 wt% NaOH solution were prepared for pH adjustment. By using a peristaltic pump, the reactant solutions were added to a three-necked flask at 30 °C according to the feeding sequence and flow rate set by the experimental design. The mixed solution was continuously stirred at 30 °C for 3 h. The pH of the solution during the whole synthesis process was kept at 11.6 ± 0.1 by the prepared HNO_3_ solution and NaOH solution. As agglomeration could occur after synthesis, the obtained C-S-H/PCE nanocomposite suspension was subjected to ultrasonic treatment with varied time of treating (15 s to 30 min).

### 2.4. Characterization

PSD of the C-S-H/PCE nanocomposites was determined by laser granulometry (LS230, Beckman Coulter, CA, USA). The C-S-H/PCE nanocomposite suspension samples were diluted with water and then tested directly or after ultrasonic treatment for a certain period of time.

Crystalline phases of the C-S-H/PCE nanocomposites were analyzed by XRD (DX-2700BH, Haoyuan Instrument Co. Ltd., Dandong, China). The C-S-H/PCE nanocomposites powder was obtained by centrifuging at 10,000 rpm for 30 min, washed with CO_2_-free deionized water, and then freeze-dried for 48 h. The analysis was performed on the powder with a Cu anode (40 kV and 40 mA), and the scanning range was from 15° to 65° 2θ with a scanning speed of 3°/min.

Morphology of the C-S-H/PCE nanocomposites was characterized by TEM (JEM-2100F, JEOL, Tokyo, Japan) and SEM (TM4000Plus, Hitachi, Tokyo, Japan). The freeze-dried powder sample was ultrasonically dispersed in anhydrous ethanol for 15 min, and then a small amount of the supernatant was placed on the copper mesh supporter, which was taken for the TEM test after two days of drying. A small amount of freeze-dried powder was laid on the sample table, and an SEM test was carried out after spraying gold.

The Roman spectra were recorded using a laser confocal micro Raman spectrometer (LABIAN HR-EVOLUTION, Horiba, Paris, France) excited by a 532 nm line to characterize the degree of polymerization of C-S-H/PCE. The preparation method of the powder sample was the same as that of the XRD sample.

## 3. Results

### 3.1. Particle Size

#### 3.1.1. Effects of Synthetic Variables on the PSD of C-S-H/PCE Nanocomposites

**Figure 1:**
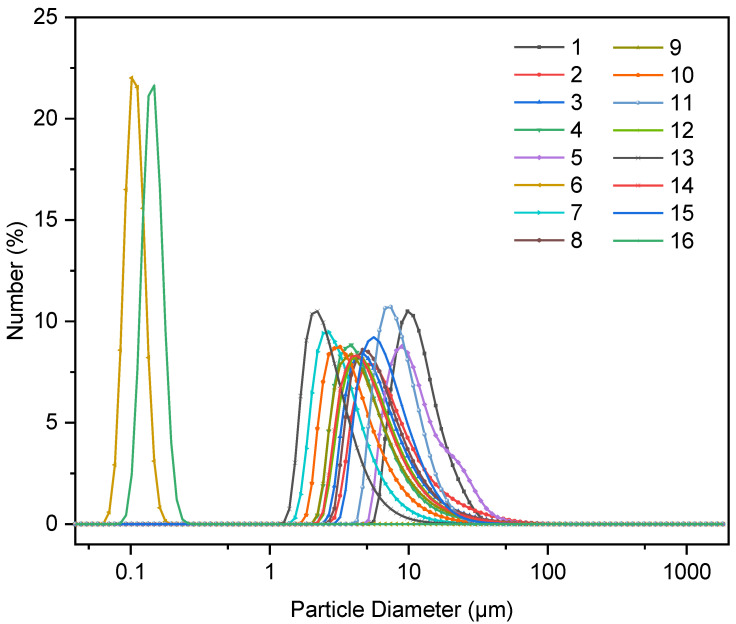
Particle size distribution of the synthesized calcium silicate hydrate/polycarboxylate (C-S-H/PCE) nanocomposites.

As shown in Figure 1, the PSD of all the 16 groups of synthesized C-S-H/PCE nanocomposites presents a single peak distribution. The PSD of two out of 16 groups varied in the range of the nanometer scale so that the median size of group 6 was 111 nm while the median size of group 16 was 150 nm. In contrast, the PSD of the other groups was in the range of the micron scale so that the median size ranged from 2 μm to 12 μm.

#### 3.1.2. Effects of Ultrasonic Treatment on the PSD of C-S-H/PCE Nanocomposites

**Figure 2:**
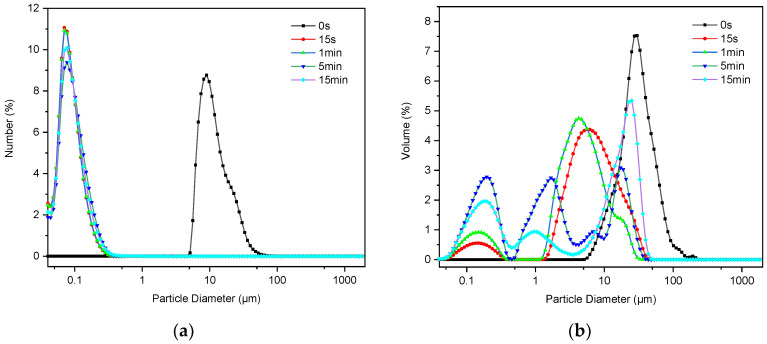
Particle size distribution of group 5 in (**a**) number and (**b**) volume.

**Figure 3:**
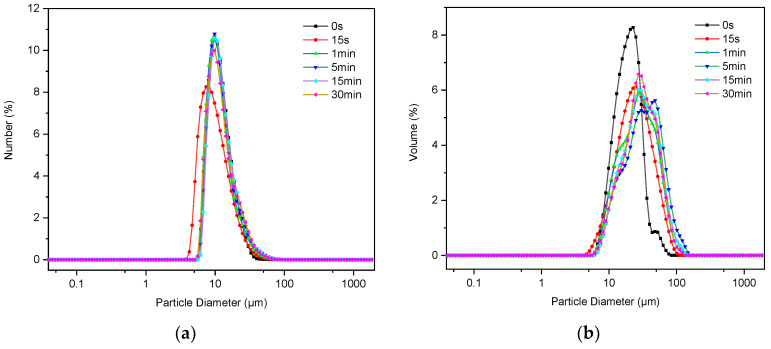
Particle size distribution of group 1 in (**a**) number and (**b**) volume.

After ultrasonic treatment, the change in the particle size of C-S-H/PCE nanocomposites showed two distinctive trends. The PSD of some groups (2, 5, 11, and 15) changed by orders of magnitude after ultrasonic treatment for a certain time that the median particle size decreased from micron to nanoscale, as represented by a typical group 5 (Figure 2a). Further analysis on the PSD in terms of volume revealed that the single peak distribution transformed into bimodal or even multi-peak distribution (Figure 2b), which indicates that large particles turned into smaller ones by ultrasound. In contrast, the PSD of other groups changed negligibly after ultrasonic treatment, as represented by a typical group 1 (Figure 3a). In most cases, C-S-H nano-particles tended to agglomerate due to their large specific area. With the aid of ultrasonic treatment, the aggregates of these nanoparticles can be broken up and then well dispersed. However, it is difficult to obtain smaller particles, even treated by ultrasound for a long time, if the agglomeration structure of the aggregates is stable. The phenomenon is highly in line with the results reported by Luc Nicoleau [24].

According to the results from ultrasonic treatment on groups 2, 5, 11, and 15, it was found that the optimal treatment time is 5 min, beyond which the change in PSD was insignificant. Therefore, the median size of synthesized C-S-H/PCE nanocomposites after ultrasonic treatment for 5 min was used for further analysis in the following sections.

#### 3.1.3. Analysis of PSD Based on the Orthogonal Experiment

Since a smaller particle size of synthesized C-S-H/PCE nanocomposites, especially in nano size, favors the seeding of the particles to be effective in cement-based materials, an index *S*, which was calculated as a reciprocal of the median particle size, was employed to quantitatively assess the influence of synthetic variables on the particle size, as shown in Table 3.

For the four levels of the feeding sequence of reactants, the *K* value of level a is much less than that of other levels. Thus, an addition of calcium and siliceous to the PCE solution is not recommended for the preparation of C-S-H composites with smaller particle sizes. Increasing the flow rate of reactants resulted in a decrease in particle size until the flow rate was higher than 0.8 mL/min, as indicated by the *K* value. The addition of reactants with a speed that is too fast is not beneficial for the formation of C-S-H/PCE nanocomposites. This is in line with the work of Sun et al. [22]. For the concentration of reactants, the *K* values of levels a and b with lower concentrations are 1~2 orders of magnitude greater than that of levels c and d with higher concentration, indicating that lower concentration of reactants helped to produce C-S-H with smaller particle size. A sharp increase was observed for the *K* value when the dosage of PCE increased from 5% to 15%, beyond which the *K* value decreased incrementally. Therefore, the contribution of PCE to the decline of particle size diminished if the concentration of PCE was higher than 15%. The *K* values in Table 3 indicate that the optimal scheme to obtain a smaller particle size of the nanocomposites is A_b_ B_c_ C_b_ D_b_.

The analysis of variance results is listed in Table 4. It is evident that the concentration of reactants had a significant impact on the particle size while other variables had an insignificant impact on the particle size. As stated earlier, C-S-H/PCE nanocomposites with smaller particle sizes could be synthesized at lower concentrations of reactants. This could be reasoned by the fact that the concentration of reactants will affect the reaction rate, which in turn affects the formation of the C-S-H. Firstly, a high reaction rate, which derives from a high concentration of reactants, would result in the fast growth of C-S-H particles. Secondly, the concentration of C-S-H generated locally is relatively high. Thus, based on the proximity effect of nanoparticles, it can be inferred that C-S-H particles are more likely to agglomerate and form stable aggregates.

Compared with the feeding sequence of reactants and the flow rate of reactants, the dosage of PCE had a relatively higher significance. The addition of PCE could help to disperse the C-S-H particles and prevent them from agglomeration. However, a further increase in the dosage of PCE, e.g., 15% in this study, could result in redundancy of the dispersant. Sun et al. [22] also showed that C-S-H with smaller particle sizes could be obtained when the PCE dosage was the intermediate value. In addition, Fang Wang et al. [37] and Kanchanason et al. [41] showed that the performance of PCE also had a significant impact on the particle size of C-S-H.

### 3.2. Composition, Microstructure, and Morphology

#### 3.2.1. XRD

**Figure 4:**
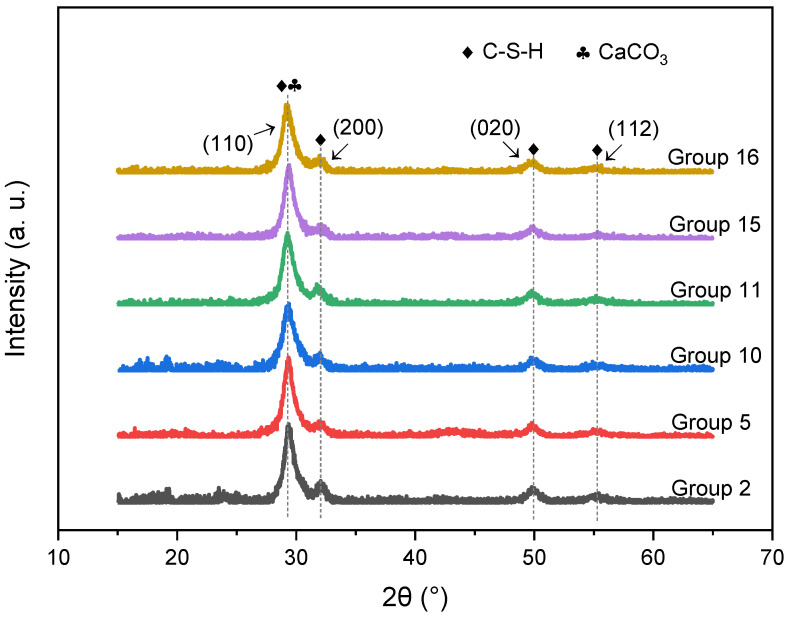
X-ray diffraction patterns of 6 groups of the C-S-H/PCE nanocomposites.

In this work, 6 out of 16 groups of C-S-H/PCE samples were selected for XRD analysis, as shown in Figure 4. Basically, no difference was observed in diffraction patterns among the groups. Thus, the change in synthetic variables had little influence on the composition of the products. Four characteristic peaks near 29°, 32°, 50°, and 55°, corresponding to (110), (200), (020), and (112), respectively, crystal planes of the product C-S-H, were identified. However, the widening of these peaks indicates a low crystallinity degree of the C-S-H. Peaks derived from calcium carbonate were also detected, indicating slight carbonization during the synthesis process.

Based on the XRD results, the relative crystallinity was calculated by the peak fitting method, as shown in Table 5. The highest and the lowest relative crystallinity were achieved in group 16 and group 10, respectively. It is interesting to note that nano-sized particles could be directly obtained without treatment for group 16, while it was quite difficult to obtain nano-sized particles even subjected to a long time of ultrasonic treatment for group 10. This phenomenon indicates that there is a correlation between the particle size and the relative crystallinity of the synthesized C-S-H. According to the precipitation mechanism, it tends to form amorphous precipitation when the aggregation rate of the crystal nucleus is greater than the orientation rate. The aggregation rate is highly dependent on the supersaturation of the solution, which can be achieved with a high concentration of reactants. Therefore, it is reasonable that the crystallinity of group 10 with the highest concentration of reaction solution would result in a high supersaturation, which in turn leads to a high aggregation rate and precipitation with the lowest crystallinity.

#### 3.2.2. SEM/TEM

**Figure 5:**
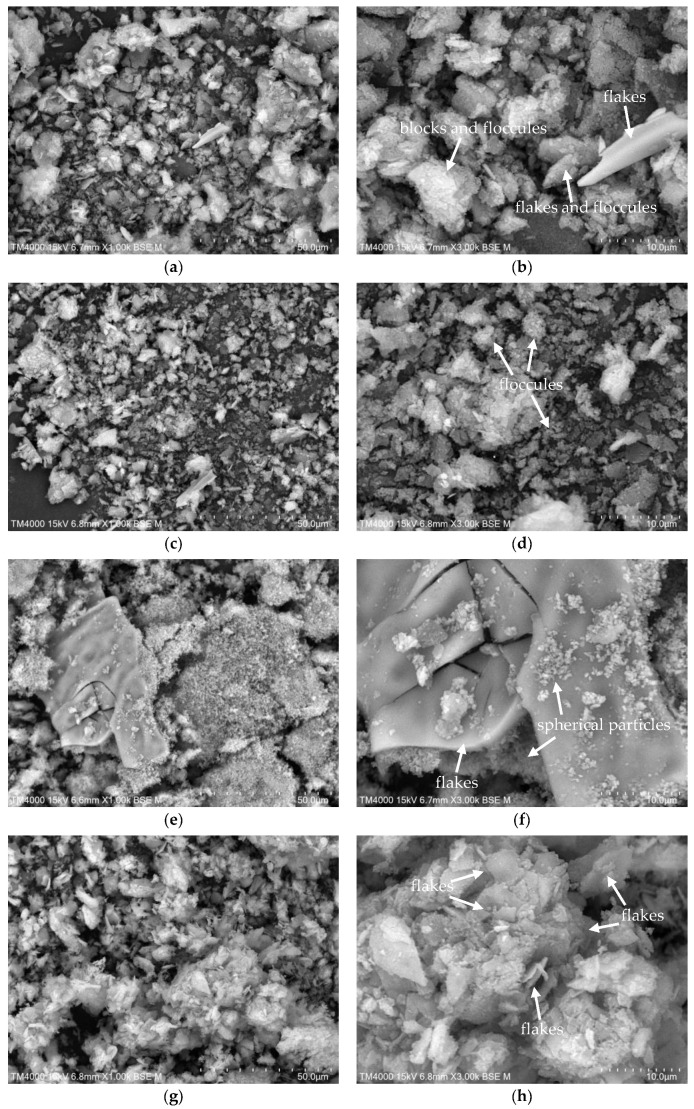
Scanning electron microscopy images of 4 groups of C-S-H/PCE nanocomposites. (**a**) Group 2, 1000× magnification. (**b**) Group 2, 3000× magnification. (**c**) Group 6, 1000× magnification. (**d**) Group 6, 3000× magnification. (**e**) Group 10, 1000× magnification. (**f**) Group 10, 3000× magnification. (**g**) Group 15, 1000× magnification. (**h**) Group 15, 3000× magnification.

Morphology of 4 groups (2, 6, 10, and 15) was selected for SEM analysis, as shown in Figure 5. Under 1000× magnification, it could be seen that the particle size of group 6 was significantly smaller than groups 2 and 15, and the particle size of group 10 was the largest. However, it was difficult to distinguish the specific size in each group due to the disorderly accumulation or agglomeration. The variety of the morphology among some groups could be noticed under 3000× magnification. Irregular blocks with some floccules attached and a small number of irregular flakes were observed in group 2 (Figure 5b). Mostly floccules, without obvious edges and angles, were piled up disorderly in group 6 (Figure 5d). Large dense flakes with small spherical particles attached were noticed for group 10 (Figure 5f). A large number of irregular flakes were disorderly and densely stacked for group 15 (Figure 5h).

The varied synthetic variables, especially the concentration of reactants, could be responsible for the difference in the morphology of C-S-H/PCE nanocomposites. The lowest and the highest concentration of reactants was found in group 6 (level a) and group 10 (level d), respectively, while groups 2 and 15 had the same level of concentration of reactants (level b). Groups with a low concentration of reactants tended to form floccules with small grain sizes. With the increase in the concentration of reactants, the products began to show flakes or blocks of relatively large size. A further increase in the concentration of reactants resulted in large and dense flakes, which could be attributed to the agglomeration. The agglomerates formed in a very stable structure, so the particles were difficult to separate even with a long ultrasonic treatment time. The difference in the morphology between groups 2 and 15 was possibly caused by the different dosages of PCE dispersant. Group 15 with higher PCE content tended to form flakes with a relatively lower degree of agglomeration.

**Figure 6:**
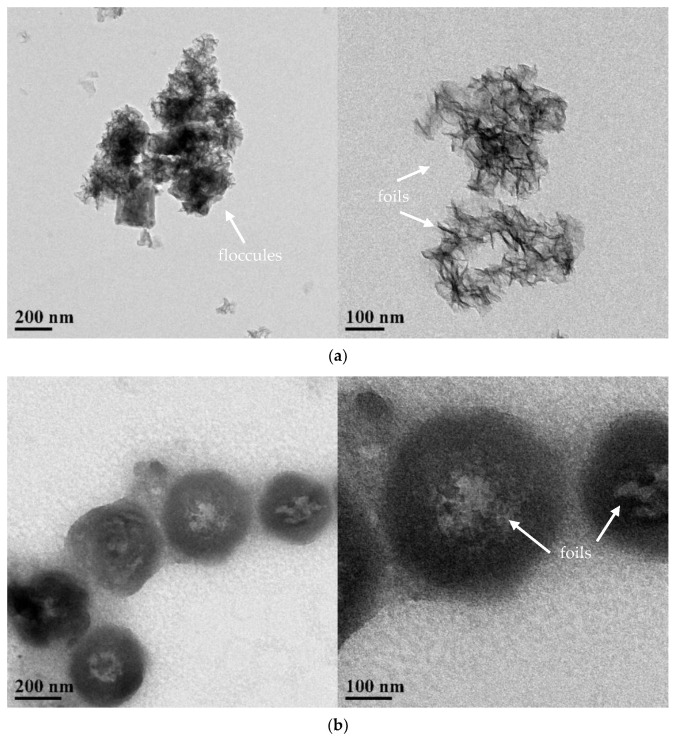

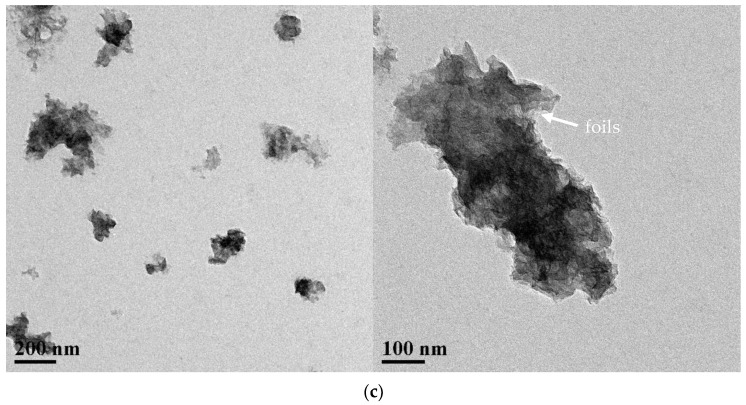
Transmission electron microscopy images of 3 groups of C-S-H/PCE nanocomposites. (**a**) Group 2. (**b**) Group 10. (**c**) Group 15.

TEM results on groups 2, 10, and 15 are shown in Figure 6. Both group 2 and group 15 showed flocculent structures with different sizes of particles formed by foil agglomeration (Figure 6a,c), which was similar to the floc observed in the SEM images (Figure 5). The large difference in the size of the flocculent aggregates could be due to an uneven dispersion of the particles. In contrast, group 10 showed regular spheres connected in chains (Figure 6b). The size of the spherical particles is relatively uniform. Moreover, foils were identified at the center of the spheres, indicating that these spherical particles were probably formed by the agglomeration of foils, which were found in groups 2 and 15. The difference in the morphology between group 10 and other groups could be attributed to the varied mode of agglomeration of particles. Plank et al. [25] also reported the phenomenon of spheres transforming into nanofoils for synthetic C-S-H.

#### 3.2.3. Raman Spectrum

**Figure 7:**
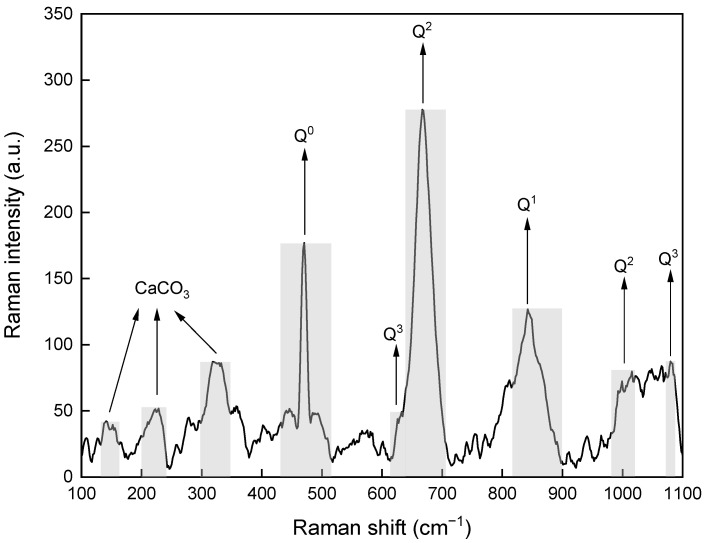
Raman spectrum of group 5 of C-S-H/PCE nanocomposites.

Since a similar pattern was obtained for all groups, a representative Raman spectrum of group 5 was presented (Figure 7). There were multiple peaks less than 320 cm^−1^ that were probably due to the vibration Ca-O polyhedral, indicating the presence of CaCO_3_. This is consistent with the XRD results. The peak at ~470 cm^−1^ was primarily attributed to internal deformations of the SiO_4_ tetrahedra. In the Si–O–Si symmetrical bending region, there was a broad band in the range from 615 cm^−1^ to 700 cm^−1^ and is assigned to linkages involving Q^2^ and Q^3^ tetrahedra. Another broad band at ~ 840 cm^−1^ caused by symmetrical stretching of Si-O was assigned to Q^1^ sites. The peaks at ~1000 cm^−1^ and ~1080 cm^−1^, which were also caused by symmetrical stretching of Si-O, could be assigned to Q^2^ and Q^3^ tetrahedra, respectively. It can be noted that the signal of Q^1^ is considerably lower than the signal of Q^2^. According to John et al. [42], The mean chain length of silicate, which was calculated from the Q^1^ to Q^2^ ratio, decreased with an increasing Ca/Si. When the Ca/Si was higher than 1.2, most of the silicate chains were dimeric. Since the Ca/Si was fixed at 1.5, a low degree of silicate polymerization can be expected in this study.

## 4. Conclusions

- The concentration of reactants had a significant effect on the particle size of the synthesized C-S-H/PCE, followed by the dosage of dispersant, while the feeding sequence and flow rate of reactants had little impact on the particle size. Low concentration of reactants and sufficient dispersant content are conducive to the synthesis of C-S-H/PCE with nano median size;
- Ultrasonic treatment for 5 min could effectively break the unstable structure of nano C-S-H/PCE aggregates. However, it had a negligible effect on the stable nano C-S-H/PCE aggregates, even if the treatment time was extended to 30 min;
- The change in variables had little influence on the composition of the synthesized C-S-H/PCE nanocomposites but had a significant influence on their crystallinity and morphology. Low concentrations of reactants tended to form products with high crystallinity. The concentration of reactants affected the morphology of the nanocomposites so that low concentrations of reactants tended to form incompact floccules, while high concentrations of reactants tended to form relatively dense flakes or blocks.

## Figures and Tables

**Table 1:** Variables and levels selected for the orthogonal design.

Levels	A	B	C	D
	Feeding Sequence of Reactants	Flow Rate of Reactants	Concentration of Reactant Solution	Dosage of PCE
a	Calcium and siliceous were added to the PCE solution	0.2 mL/min	Ca(NO_3_)_2_: 0.1 mol/L Na_2_SiO_3_: 0.05 mol/L	5%
b	Calcium was added to a mixture of silica and PCE	0.5 mL/min	Ca(NO_3_)_2_: 1 mol/L Na_2_SiO_3_: 0.5 mol/L	15%
c	Silica was added to a mixture of calcium and PCE	0.8 mL/min	Ca(NO_3_)_2_: 3 mol/L Na_2_SiO_3_: 1.5 mol/L	30%
d	Calcium, siliceous, and PCE were added simultaneously	1.1 mL/min	Ca(NO_3_)_2_: 7 mol/L Na_2_SiO_3_: 3.5 mol/L	50%

**Table 2:** L_16_(4^5^) orthogonal arrangement.

Groups	A	B	C	D
1	a	a	a	a
2	a	b	b	b
3	a	c	c	c
4	a	d	d	d
5	b	a	b	c
6	b	b	a	d
7	b	c	d	a
8	b	d	c	b
9	c	a	c	d
10	c	b	d	c
11	c	c	a	b
12	c	d	b	a
13	d	a	d	b
14	d	b	c	a
15	d	c	b	d
16	d	d	a	c

**Table 3:** *K* value analysis of the results from the orthogonal experiments.

Groups	A	B	C	D	Median Particle Size, μm	*S*, μm^−1^
1	a	a	a	a	11.66	0.09
2	a	b	b	b	0.08	11.78
3	a	c	c	c	5.91	0.17
4	a	d	d	d	4.77	0.21
5	b	a	b	c	0.08	11.90
6	b	b	a	d	0.11	8.99
7	b	c	d	a	3.27	0.31
8	b	d	c	b	6.17	0.16
9	c	a	c	d	4.87	0.21
10	c	b	d	c	3.94	0.25
11	c	c	a	b	0.06	16.43
12	c	d	b	a	5.48	0.18
13	d	a	d	b	2.66	0.38
14	d	b	c	a	5.33	0.19
15	d	c	b	d	0.09	11.55
16	d	d	a	c	0.15	6.68
*K*_a_^1^	12.25	12.56	32.19	0.76	Optimal scheme	
*K*_b_^1^	21.35	21.21	35.41	28.75		
*K*_c_^1^	17.08	28.46	0.72	19.00	A_b_ B_c_ C_b_ D_b_	
*K*_d_^1^	18.80	7.23	1.15	20.95		

^1^*K*_i_ represents the sum of the corresponding test results when the level number of the factor is i. The larger the sum, the more favorable the synthesis of C-S-H with small particle size.

**Table 4:** Analysis of variance.

Source of Variance	Sum of Squares of Deviations	Degree of Freedom	Sum of Squares of Mean Deviations	F	Critical Value	Significance
A	11.057	3	3.686	0.20		
B	65.899	3	21.966	1.21		
C	271.320	3	90.440	4.97	F_0.05_(3,15) = 3.29	* ^2^
D	105.219	3	35.073	1.93		
Test error	54.616	3	18.205			
Sum	508.111	15				

^2^ When the F value of the factor is greater than F_0.05_(3,15), it means that the factor has a significant influence on the particle size of the synthesized C-S-H, which is represented by the symbol *.

**Table 5:** The calculated relative crystallinity of the C-S-H/PCE nanocomposites.

Groups	2	5	10	11	15	16
Relative crystallinity/%	91.14 ± 1.43	91.46 ± 1.49	85.62 ± 1.36	90.62 ± 1.47	90.42 ± 1.69	94.30 ± 1.52

## Data Availability

Not applicable.
